# Personal involvement is related to increased search motivation and associated with activity in left BA44—a pilot study

**DOI:** 10.3389/fnhum.2015.00144

**Published:** 2015-03-26

**Authors:** Michael Schaefer, Franziska Rumpel, Abdolkarim Sadrieh, Martin Reimann, Claudia Denke

**Affiliations:** ^1^Department of Neurology, Otto-von-Guericke University MagdeburgMagdeburg, Germany; ^2^Department of Marketing, Otto-von-Guericke University MagdeburgMagdeburg, Germany; ^3^Department of E-Business, Otto-von-Guericke University MagdeburgMagdeburg, Germany; ^4^Eller College of Management, University of ArizonaTucson, AZ, USA; ^5^Department of Anesthesiology and Intensive Care Medicine, Charité—Universitätsmedizin BerlinBerlin, Germany

**Keywords:** involvement, decision behavior, social neuroscience, fMRI

## Abstract

Numerous studies explore consumer perception of brands in a more or less passive way. This may still be representative for many situations or decisions we make each day. Nevertheless, sometimes we often actively search for and use information to make informed and reasoned choices, thus implying a rational and thinking consumer. Researchers suggested describing this distinction as low relative to high involvement consumer behavior. Although the involvement concept has been widely used to explain consumer behavior, behavioral and neural correlates of this concept are poorly understood. The current study aims to describe a behavioral measure that is associated with high involvement, the length of search behavior. A second aim of this study was to explore brain activations associated with involvement by employing functional magnetic resonance imaging (fMRI). We presented participants information cues for different products and told them that they had to answer questions with respect to these products at the end of the experiment. Participants were free to stop the information search if they think they gathered enough information or to continue with collecting information. Behavioral results confirmed our hypothesis of a relationship between searching behavior and personal involvement by demonstrating that the length of search correlated significantly with the degree of personal involvement of the participants. fMRI data revealed that personal involvement was associated with activation in BA44. Since this brain region is known to be involved in semantic memory, the results of this pilot study suggest that high involvement consumer behavior may be linked to cognitive load and attention towards a product.

## Introduction

It is well known that there often exist strong emotional bonds between a brand and the customer (Engle and Blackwell, 1982; Leahy, 2008). For example, it has been demonstrated that the perception of favorite brands or products involves similar brain networks as artificially associated reward stimuli (e.g., O’Doherty, 2004; O’Doherty et al., 2006; Knutson et al., 2007; Schaefer and Rotte, 2007a; Schaefer et al., 2011). This has been explained by the association of brands with appetitive stimuli, e.g., due to marketing efforts (Gorn, 1982; Leahy, 2008). However, when we face brands or consumer products we are not only driven by emotional and unconscious factors. Sometimes we actively and extensively search for information to make informed and reasoned choices. Thus, the consumer here is a rational and intelligent individual, looking extensively for information and performing a comprehensive evaluation of the choice alternatives. It has been proposed that this attitude can be described as high or, respectively, as low involvement consumer behavior, which can be differentiated, for example, by the degree in which consumers actively and extensively search for information (Zaichkowsky, 1985). However, the involvement construct is complex and has many different facets. Antecedents of involvement may be personal factors (needs, values, importance), stimulus factors (differentiation of alternatives, content of communication), as well as situational factors (occasion, purchase/use). Hence, one can be involved with products, advertisements, or with purchase decisions. Possible results of involvement may be the amount of information search, the effectiveness of an ad to induce purchase, the relative importance of the product class, the perceived differences in product attribution, the preference of a particular brand, the influence of price on brand choice, and the time spent deliberating alternatives (Zaichkowsky, 2012). Given the complexity of this concept Kassarjian (1981) claimed that it was “unfortunate that a simple instrument or tool has not yet been developed to measure the concept of involvement but if ‘necessity is the mother of invention’ that will come in time—for the measure of levels of involvement is unquestionably a necessity—one that can no longer be ignored”. This need was addressed 1985 by Judith Lynne Zaichkowsky, who developed a bipolar adjective scale to measure the concept of involvement (Personal Involvement Inventory, Zaichkowsky, 1985, 1994). In the following years this instrument has been widely used to examine consumer behavior in various contexts.

Although the concept of involvement and the Personal Involvement Inventory resulted in numerous studies, little is known about behavioral correlates of this distinction. One important result of this concept is that search behavior in advance to a decision should differ with respect to this distinction. Thus, high involvement should be associated with an increased length of search behavior when collecting data. The present experiment explores behavioral correlates for search behavior in order to test this hypothesis. We assumed that collecting information for high involvement products results in increased search behavior (relative to low involvement products).

A second aim is to examine neural correlates for involvement. Recent advances in neuroimaging now allow examining theoretical constructs beyond behavioral dimensions, providing information with respect to their underlying neuronal networks. Here we aimed to test this dichotomy of consumer behavior by means of functional magnetic resonance imaging (fMRI). Given that involvement can be seen as the personal meaning of a specific object in a certain situation (Zaichkowsky, 1985), we hypothesized that high involvement consumer behavior is associated with brain regions known to be related to self-related cognitions. Those brain areas are the precuneus (e.g., Cavanna and Trimble, 2006) and the prefrontal cortex (e.g., Johnson et al., 2002; Ochsner et al., 2004; Schaefer and Rotte, 2007b). Activation in the prefrontal part of the brain has been related to personality expression, decision-making, searching processes and planning of complex cognitive behavior. In particular, it has been related to actions with internal goals (Miller et al., 2002) and to reward expectation (Watanabe, 1996). Thus, we hypothesized that high involvement consumer behavior (relative to low involvement) is linked to increased activation in prefrontal brain regions.

In order to test both hypotheses we used a paradigm in which participants were presented information to specific products, similar to a search on the Internet with a search engine. Participants were asked to collect information for various products. They were told that after the end of the experiment a questionnaire would have to be completed, in which they were asked questions for each product and rewarded with 0.30 € for each correct answer. After each information cue the participants had to decide if they want to receive more information or to stop the information search for this product. Since the participant was not told what kind of questions we would ask him, we assumed that the length of search behavior was depending on his or her personal involvement in a product. Thus, we hypothesized that the number of information cues would be associated with the degree of personal involvement. In other words, we assumed that the more participants were personally involved in a product, the more information they would collect. We hypothesized that this involvement attitude is linked to activity in prefrontal cortex.

## Experimental Procedure

### Participants

Ten subjects (five females) with a mean age of 25 years participated in the study. All participants were students from the local university, recruited from a data pool of volunteers. Each of them received 18 Euros for participating in this study. The participants gave informed written consent to the study, which adhered to the Declaration of Helsinki and was approved by the local human subjects’ committee (University of Magdeburg).

### Procedure

Participants were instructed to collect information on different products similar to a search on the Internet. They were told that after the end of the experiment a questionnaire on those products would be presented. For each correct answer they would get 0.30 €.

**Figure 1:**
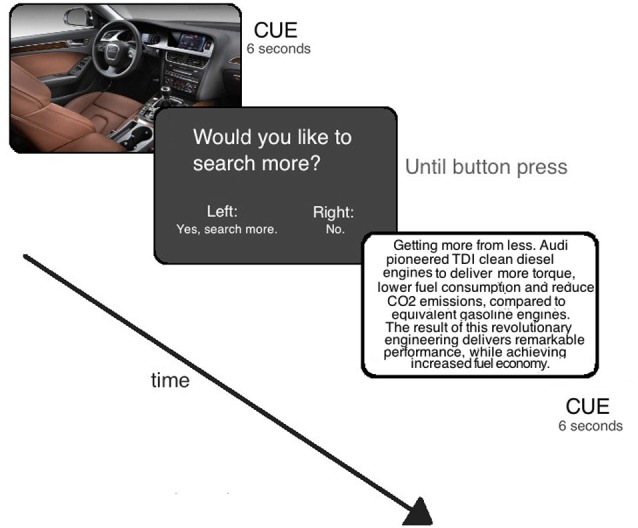
**Design of the search task**. For details see text.

Information stimuli consisted out of texts or pictures providing information on a certain product (information cues), for example, displaying the picture of a specific car model or providing information about technical features of a car (e.g., gasoline consumption) (see Figure 1). There was a total of 30 information cues for each product. Participants viewed 30 products. The stimuli were taken from a pre-study, which controlled all items to match with respect to valence. Products included objects such as cameras, cars, or cosmetics. All stimuli were non-fictional. Participants had no prior knowledge about the objects (as verified before including them in the study).

Each information cue (picture of text) lasted for 6 s and was followed by a question screen: “Would you like to search more?” Here the participants had to choose if they wanted to get more information on this product (“Yes, search more.”) or to stop the information search on this product and start with another product (“No, next product.”) (see Figure 1). If they decided to see more information the next information cue on this product would be presented. If they chose to stop the search here, an information cue on another product would show up. The question screen appeared for 2 s. Participants were allowed to spend up to 30 s to respond to this question. The next trial started 6 s after the response of the participants. Condition-related activity was measured using a “floating” time window of eight images surrounding (four before, one during, and three after) the point of response for each participant (analog to Greene et al., 2001). In addition, we applied a baseline condition, which displayed a fixation asterisk for 15 s. Participants were instructed to relax in this resting condition.

In order to respond to the questions participants had to press a key with their right hand. The key was custom-made and had two buttons.

Visual images were back-projected to a screen at the end of the scanner bed close to the subject’s feet (projector: JVC DLA G150CL). Subjects viewed the images through a mirror mounted on the birdcage of the receiving coil.

The experiment consisted of two runs. Each run included all conditions. The experiment lasted for about 1 h. Participants spent about 45 min inside and about 15 min outside the scanner (for preparing for MRI, explaining the task and completing the RPII).

After the fMRI experiment subjects were asked to complete a German version of the Personal Involvement Inventory (RPII, Zaichkowsky, 1994). The RPII is a 10-item self-report survey asking to judge various products against a series of descriptive affective and cognitive adjectives according to their personal perception. For example, participants were instructed to judge if a product is close to the adjective “boring” or “interesting” (Zaichkowsky, 1994). According to Zaichkowsky the RPII can be divided into a cognitive and in an affective scale. Participants completed the RPII for all product categories.

### fMRI Data Acquisition and Analysis

We used a 1.5 T scanner (General Electrics Signa LX, Fairfield, Conneticut, USA) to conduct functional imaging (gradient echo T2-weighted echo-planar images; TR = 2 s, TE = 35 ms, flip angle = 80°, FOV = 20 mm). Data were acquired in two functional imaging sessions. Due to T1 equilibration effects, we discarded the first three volumes of the functional data for each session. Functional volumes consisted of 23 slices. Each volume comprised 5 mm slices (1 mm gap, in plane voxel size 3.125 × 3.125 mm). Functional slices were acquired interleaved in ascending order. In order to facilitate localization and coregistration of functional data, high-resolution anatomical scans were additionally acquired using a T1-weighted spoiled gradient recalled echo sequence (TR = 520 ms, TE = 9 ms, flip = 90°).

fMRI data was preprocessed and analyzed using the Statistical Parametric Mapping Software (SPM8, Wellcome Department of Imaging Neuroscience, University College London, London, UK). For each participant the fMRI scans were realigned to correct for inter-scan movement using sinc interpolation and subsequently normalized into a standard anatomical space (MNI, Montreal Neurological Institute template), resulting in isotropic 3 mm voxels. The scans were then smoothed with a Gaussian kernel of 8 mm full-width half maximum.

Statistical parametric maps were calculated using multiple regression with the hemodynamic response function modeled in SPM (boxcar-function). First, we examined data on the individual subject level by using a fixed effects model. Second, the resulting parameter estimates for each regressor at each voxel were then entered into a second-level analysis by using a random effects model. Statistical contrasts (*t* tests) were performed to examine cortical activation associated with the first positive response (“Yes, more information”) relative to the last positive response. The last positive response was defined as the response before indicating that the participant did not want to receive more information on this product. We assumed that the first response represents a situation with high involvement, since the participant here wants to receive more information. In contrast, the last positive response represents a state with low involvement, because after the next image he will finish the information collection for this product. Furthermore, we added time (number of cues between first and last search item) as a covariate in our analysis. Finally, we correlated the percentual signal change of the BOLD responses (peak activations) with the behavioral responses (using SPM).

In addition, we used a second measure to test our hypotheses. We compared brain activation for products with long search duration (high involvement) compared with products with short search duration (low involvement).

The resulting images were thresholded at *p* < 0.05 corrected for multiple comparisons (FWE). Correction was achieved by imposing a threshold for the volume of clusters comprising contiguous voxels that passed a voxel-wised threshold of *p* < 0.001.

Anatomical interpretation of the functional imaging results was performed by using the SPM Anatomy toolbox (Eickhoff et al., 2005).

## Results

### Behavioral Results

One participant was excluded prior further data analysis due to technical reasons. None of our participants claimed to have difficulties with the task or had times when he or she felt bored or exhausted. In addition, none of our participants stated to have used any strategies in order to perform the task.

**Figure 2:**
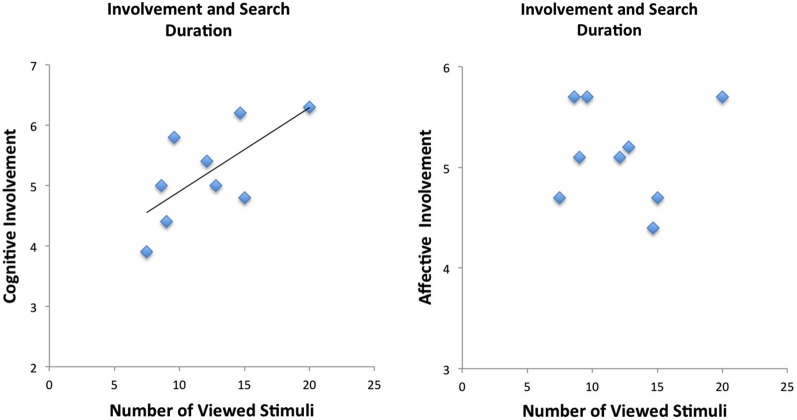
**Behavioral results demonstrate correlation between cognitive scale of the involvement but not of the affective scale of the involvement questionnaire**.

The mean number of viewed information cues was 19.36 (± 2.06 standard deviation) (total possible number of information cues was 30). Analysis of the behavioral data revealed correlations between self-reported involvement (RPII scores) and number of viewed stimuli (information cues): The number of viewed stimuli (collapsed across products for each individual) correlated significantly with the cognitive dimension of the RPII (*r* = 0.69, *p* < 0.05) (see Figure 2 and Table 1). Thus, our hypothesis that more stimuli are viewed for high involvement products received support by showing that more stimuli were viewed (on average) when participants were more involved with products (in general). Correlation with the affective dimension failed to reveal significant activations (*p* > 0.10). The affective and the cognitive dimensions of the RPII were not significantly correlated (*r* = 0.28, *p* = n.s.).

**Table 1:** **Mean number of cues (pictures and text cues) between first and last responses for all participants (across all products)**.

Participants	Sex	Mean number of cues between first and last response
**1**	Male	15
**2**	Female	12
**3**	Male	8
**4**	Female	20
**5**	Female	15
**6**	Male	10
**7**	Female	9
**8**	Male	12
**9**	Female	13
**10**	Male	9

Statistical comparison of the reaction times between first and last positive response did not show significant results.

In addition, we examined if picture and text cues were not equally distributed across the categories first and last responses. Statistical comparisons revealed no significant differences.

### fMRI Results

**Figure 3:**
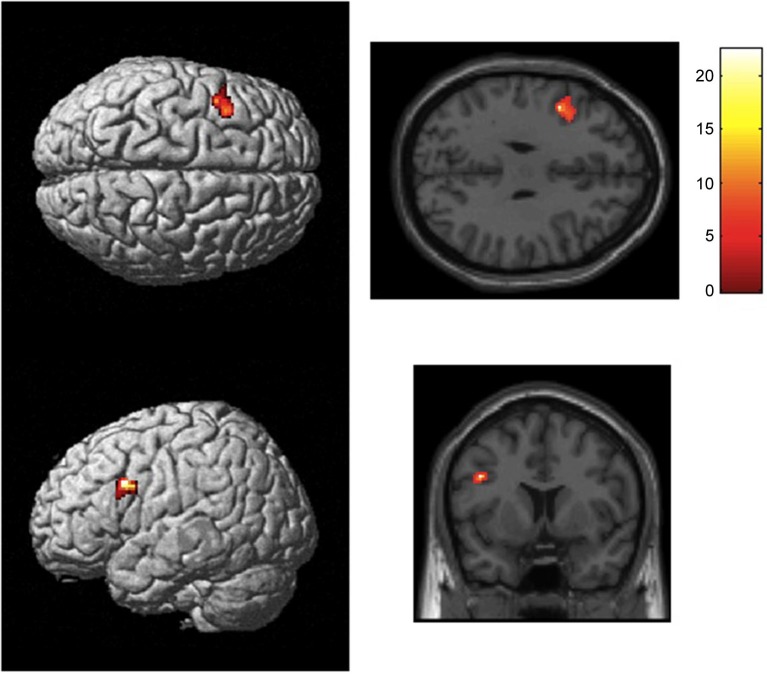
**Statistical map showing brain activation for the first positive response (“Yes, more information”) relative to the last positive response (random-effects analysis**, *p*** < 0.05, FWE corrected)**. Results revealed activation of left BA44. Areas of significant fMRI signal change are shown as color overlays on the T1-MNI reference brain.

Analysis of the fMRI data for the BOLD activity during the first relative to the last positive response revealed brain activation in left inferior frontal gyrus (BA44, pars opercularis, peak MNI coordinates: −44, 8, 30, *z* = 5.35, 158 voxels, FWE corrected). Furthermore, the left middle frontal gyrus, the left inferior temporal gyrus, and the right angular gyrus were involved (see Table 2 and Figure 3). Comparison of brain activity during last positive response relative to first positive response failed to reveal significant activations. Analysis of the covariate time revealed no significant results.

**Table 2:** **Results of random effects analysis for brain activation for the first positive response (“Yes, more information”) relative to the last positive response (before stopping the search) (random-effects analysis, *p* < 0.05, FWE corrected, L = left hemisphere, R = right hemisphere, in brackets: uncorrected results)**.

Contrast	Brain region	Peak MNI location (*x, y, z*)	Peak *z*-value	Number of voxels
first response > last response	L inferior frontal gyrus (BA44, pars opercularis)	−44, 8, 30	5.35	158
	(L middle frontal gyrus)	−24, 2, 46	4.48	91
	(L inferior temporal gyrus)	−54, −56, −12	3.73	10
	(R angular gyrus)	38, −54, 24	3.53	6
	cerebellum	30, −66, −36	3.86	50
last response > first response	-	-	-	-

Analysis of the brain responses for products with long search duration (high involvement) compared with products with short search duration revealed no significant results (at *p* < 0.05, FWE corrected). However, when using the results of our first measure as a region of interest, we found a significant activation (peak MNI coordinates of this cluster −32, 0, 34, *z* = 4.21, FWE corrected, small volume correction (SVC)).

### Combination of Behavioral and fMRI Data

**Figure 4:**
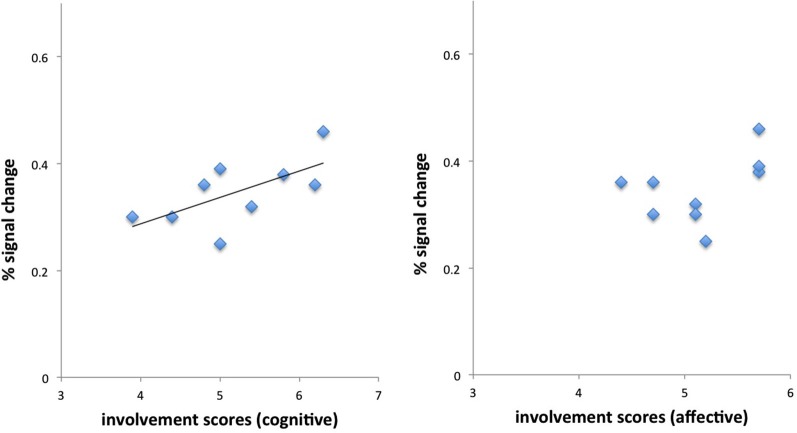
**Correlations of cognitive and affective dimensions of the involvement questionnaire with brain activations in BA44**. Only the cognitive dimension demonstrated a positive significant relationship with BOLD responses in BA44. See text for details.

We then correlated individual’s differential brain activations for the contrast first relative to last response with individual RPII scores. Results demonstrated a positive correlation of brain responses with the cognitive scale of the involvement questionnaire in left BA44 (peak MNI coordinates: −44, 8, 30, *r* = 0.64, *p* < 0.05, Pearson), but no correlation with the affective scale (*r* = 0.45, *p* = n.s.) (see Figure 4). Furthermore, BOLD response in BA44 was associated with the number of viewed information cues over all products (*r* = 0.52).

Correlations of individual RPII scores with other brain regions (left middle frontal gyrus, left inferior temporal gyrus, right angular gyrus) failed to reach the level of significance.

## Discussion

Consumer behavior can be very different. On the one hand we sometimes actively and extensively search for information to make reasoned choices. On the other hand, our consumer behavior is often passive, intuitive, and non-conscious. Zaichkowsky (1985) suggested describing these different kinds of consumer behavior with the involvement concept, which can be measured by a semantic differential introduced by Zaichkowsky (Osgood et al., 1957; Zaichkowsky, 1985, 1994). Here we aimed to examine behavioral correlates as well as the underlying neural correlates for this concept. Behavioral results confirmed our hypothesis by demonstrating that high involvement consumer behavior is associated with search duration. Furthermore, fMRI data showed that personal involvement is linked to activation in left BA44.

Involvement theory has become an important concept in the consumer behavior literature. Since behavioral correlates of high involvement are often described as longer and sustained search behavior (Zaichkowsky, 1985), we here conducted a study in which participants performed a searching task. This searching task included pictures and text information about different products. We hypothesized that high involvement products were followed by longer search duration. Results confirmed our hypothesis by showing that RPII scores were significantly correlated with the length of the search. Thus, our results provide behavioral evidence for the involvement concept. However, the correlation with behavioral results was valid only for the cognitive scale, the affective scale does not seem to be related to the length of searching behavior.

In addition, we found brain regions associated with searching behavior. Results revealed that a more sustained searching behavior was linked predominantly to an activation of the left BA44. Moreover, our results demonstrated that activity in this brain region correlated with the personal involvement of the participants. Thus, the more the participants were personally involved in the objects, the more this prefrontal brain region was engaged during the searching task. However, again only the cognitive dimension of the involvement questionnaire was linked to brain activation in BA44, the affective dimension failed to reach the level of significance.

It is well known that BA44 is in particular associated with language functions (Broca-area). Furthermore, BA44 (left IFG) has been related to semantic memory processes. For example, Wagner et al. (1998) demonstrated that the ability to later remember a verbal experience is predicted by the magnitude of activation in left IFG. In the current study our results suggest that the activation in this brain region point to an increased cognitive load and improved attention reflecting the high involvement attitude. Furthermore, recent work also suggests a role for the left IFG in risk processing (Fecteau et al., 2008; Christopoulos et al., 2009). Since high involvement is also known to occur when an individual expects a purchase with a high level of perceived risk (which can be financial but also social risk), the brain imaging data is also in line with this risk facet of the involvement concept.

fMRI results also suggest a role for the right angular gyrus (although significant only at an uncorrected level). Previous research linked this brain region to complex language functions. For example, lesions of this part of the brain result in alexia or agraphia (Gerstmann, 1940). Furthermore, our results include activation of left inferior temporal gyrus during task performance. This brain region is suggested to work together with other brain regions in order to process and recognize information of “what” something is (the ventral stream of two-stream hypothesis, Goodale and Milner, 1992). Both explanations are in line with requirements of the given task in the current experiment.

The present results demonstrate relationships of the cognitive scale of the RPII with length of search behavior as well as with brain activations in BA44. The affective scale of the RPII failed to reveal any relationships with behavioral and neuroimaging data. So why is only the cognitive scale linked to our results? It seems that the affective dimension of the RPII should be linked to other concepts than search behavior. One could speculate that there may be two different ways of being involved rather than one global concept of personal involvement. However, given that this is the first study suggesting behavioral correlates for the involvement concept, we think that future research is needed in order to proof this hypothesis and rule out that other tasks may affect both the affect and cognitive scales.

There may be several objections to our study. Both our behavioral as well as our fMRI data may reflect predominantly attention processes. However, it seems very difficult to fully control for attentional processes when experimentally investigating the involvement concept. In fact, attention seems to be a result of involvement. Another concern may focus on the fact that last and first responses differed with respect to the memory load. Hence, memory effects might have affected our findings. Nevertheless, since some of our participants viewed a relatively large number of cues for high involvement products, we think it is unlikely that memory effects may explain our results. In addition, the positive correlation between brain responses and the cognitive scale of the RPII may simply result from the fact that subjects searched longer when they were highly involved in a product. However, since there is a correlation with the RPII (and not only with the number of shown cues) we believe that this explanation is unlikely. Furthermore, we included time (number of cues) in our analysis as a covariate, which resulted in no significant results. Last, the search task was not very strictly designed. Thus, participants might have been unsure what kind of information they should look for. However, the basic idea of this experiment was to examine neural correlates for different degrees of involvement rather than for searching behavior in general. The idea was to give the participants this only roughly defined aim of the searching task in order to gather information on the personal involvement concept. Hence, we believe that this task was appropriate.

However, there are some limitations of our study. First, the sample of the present study included only few participants. A bigger sample may have revealed additional brain areas associated with the involvement variable. Thus, future studies including more participants are needed to confirm the results of this pilot study. Second, the current experiment presented only a limited choice of different products. Future studies should include a higher number of participants and a bigger variety of products in order to increase the variance of different involvement behavior. Third, we are aware of the fact that the prefrontal cortex is engaged in a lot of different functions, which is limiting the possible conclusions we can draw out of the imaging results. Future studies are necessary in further investigate the relationship between the prefrontal cortex and personal involvement. However, given that the present study is the first attempt to investigate this model with a neuroimaging approach, we believe that the results are encouraging to further link economic theories with brain imaging techniques in order to understand consumer behavior. Last, our correlational analyses refer to an involvement score collapsed over different products, therefore limiting our conclusion here. However, the present results suggest that one consequence of high involvement may be the amount of information search, which is in accordance with theoretical assumptions (Zaichkowsky, 2012). In addition, our results suggest that the higher participants were involved in the shown products (on average), the higher was his or her brain response in this brain region (on average). Future research based on other study designs is needed to verify these results.

In summary, this study presents two results. First, we demonstrated for the first time a behavioral paradigm that is able to measure personal involvement beyond the well-known semantic differential (Zaichkowsky, 1994). In addition, the present results suggest that personal involvement is associated with neural activity in left BA44, a brain region that is known to be involved in semantic memory, cognitive load, and attention. However, both relationships account only for the cognitive dimension of the involvement concept. The affective dimension does not seem to be linked to these kinds of tasks. Future studies are necessary to confirm the results of this pilot study.

## Conflict of Interest Statement

The authors declare that the research was conducted in the absence of any commercial or financial relationships that could be construed as a potential conflict of interest.

## References

[B1] Cavanna A. E. Trimble M. R. (2006). The precuneus: a review of its functional anatomy and behavioural correlates. Brain 129, 564–583. 10.1093/brain/awl004 16399806

[B2] Christopoulos G. I. Tobler P. N. Bossaerts P. Dolan R. J. Schultz W. (2009). Neural correlates of value, risk and risk aversion contributing to decision making under risk. J. Neurosci. 29, 12574–12583. 10.1523/JNEUROSCI.2614-09.2009 19812332 PMC2794196

[B3] Eickhoff S. B. Stephan K. E. Mohlberg H. Grefkes C. Fink G. R. Amunts K.. (2005). A new SPM toolbox for combining probabilistic cytoarchitectonic maps and functional imaging data. Neuroimage 25, 1325–1335. 10.1016/j.neuroimage.2004.12.034 15850749

[B4] Engle J. F. Blackwell R. D. (1982). Consumer Behavior. 4th Edn. Chicago: The Dryden Press.

[B5] Fecteau S. Pascual-Leone A. Théoret H. (2008). Psychopathy and the mirror neuron system: preliminary findings from a non-psychiatric sample. Psychiatry Res. 160, 137–144. 10.1016/j.psychres.2007.08.022 18599127

[B6] Gerstmann J. (1940). Syndrome of finger agnosia, disorientation for right and left, agraphia and acalculia—local diagnostic value. Arch. Neural. Psychiatry 44, 398–408 10.1001/archneurpsyc.1940.02280080158009

[B7] Goodale M. A. Milner A. D. (1992). Separate visual pathways for perception and action. Trends Neurosci. 15, 20–25. 10.1016/0166-2236(92)90344-8 1374953

[B8] Gorn G. J. (1982). The effects of music in advertising on choice behavior: a classical conditioning approach. J. Mark. 46, 94–101 10.2307/1251163

[B9] Greene J. D. Sommerville R. B. Nystrom L. E. Darley J. M. Cohen J. D. (2001). An fMRI investigation of emotional engagement in moral judgment. Science 293, 2105–2108. 10.1126/science.1062872 11557895

[B10] Johnson S. C. Baxter L. C. Wilder L. S. Pipe J. G. Heiserman J. E. Prigatano G. P. (2002). Neural correlates of self-reflection. Brain 125, 1808–1814. 10.1093/brain/awf181 12135971

[B11] Kassarjian H. H. (1981). “Low involvement: a second look,” in Advances in Consumer Research (Vol. 8), ed Monroe K. B. (Ann Arbor: Association for Consumer Research), 31–34.

[B12] Knutson B. Rick S. Wimmer G. E. Prelec D. Loewenstein G. (2007). Neural predictors of purchases. Neuron 53, 147–156. 10.1016/j.neuron.2006.11.010 17196537 PMC1876732

[B13] Leahy R. (2008). Brand loyalty in fast moving consumer good markets: the role of bonds. Intern. J. Bus. Manage. 3, 7–20 10.5539/ijbm.v3n12p7

[B16] Miller E. K. Freedman D. J. Wallis J. D. (2002). The prefrontal cortex: categories, concepts and cognition. Philos. Trans. R. Soc. Lond. B Biol. Sci. 357, 1123–1136. 10.1098/rstb.2002.1099 12217179 PMC1693009

[B17] Ochsner K. N. Knierim K. Ludlow D. H. Hanelin J. Ramachandran T. Glover G.. (2004). Reflecting upon feelings: an fMRI study of neural systems supporting the attribution of emotion to self and other. J. Cogn. Neurosci. 16, 1746–1772. 10.1162/0898929042947829 15701226

[B18] O’Doherty J. P. (2004). Reward representations and reward-related learning in the human brain: insights from neuroimaging. Curr. Opin. Neurobiol. 14, 769–776. 10.1016/j.conb.2004.10.016 15582382

[B19] O’Doherty J. P. Buchanan T. W. Seymour B. Dolan R. J. (2006). Predictive neural coding of reward preference involves dissociable responses in human ventral midbrain and ventral striatum. Neuron 49, 157–166. 10.1016/j.neuron.2005.11.014 16387647

[B20] Osgood C. E. Suci G. Tannenbaum P. (1957). The Measurement of Meaning. Urbana, IL: University of Illinois Press.

[B21] Schaefer M. Knuth M. Rumpel F. (2011). Striatal response to favorite brands as a function of neuroticism and extraversion. Brain Res. 1425, 83–89. 10.1016/j.brainres.2011.09.055 22035566

[B22] Schaefer M. Rotte M. (2007a). Favorite brands as cultural objects modulate reward circuitry. Neuroreport 18, 141–145. 10.1097/wnr.0b013e328010ac84 17301679

[B23] Schaefer M. Rotte M. (2007b). Thinking on luxury or pragmatic brand products: brain responses to different categories of culturally based brands. Brain Res. 1165, 98–104. 10.1016/j.brainres.2007.06.038 17655834

[B24] Wagner A. D. Schacter D. L. Rotte M. Koutstaal W. Maril A. Dale A. M.. (1998). Building memories: remembering and forgetting of verbal experiences as predicted by brain activity. Science 281, 1188–1191. 10.1126/science.281.5380.1188 9712582

[B25] Watanabe M. (1996). Reward expectancy in primate prefrontal neurons. Nature 381, 629–632. 10.1038/382629a0 8757133

[B26] Zaichkowsky J. L. (1985). Measuring the involvement construct. J. Consumer Res. 12, 341–352 10.1086/208520

[B27] Zaichkowsky J. L. (1994). The personal involvement inventory: reduction, revision and application to advertising. J. Advert. 23, 59–70 10.1080/00913367.1943.10673459

[B28] Zaichkowsky J. L. (2012). “Consumer involvement: review, update and links to decision neuroscience,” in Handbook of Developments in Consumer Behaviour, eds Wells V. Foxall G. (Cheltenham: Edward Elgar Publishing), 523–545.

